# Supplementary material for: Serological Investigation and Genetic Characteristics of Pseudorabies Virus between 2019 and 2021 in Henan Province of China
Source: Viruses. 2022 Jul 30;14(8):1685. doi: 10.3390/v14081685 (PMC9412869; doi:10.3390/v14081685)
Supplement: Supplementary file 1 [file viruses-14-01685-s001.zip › Figure S1.pdf]

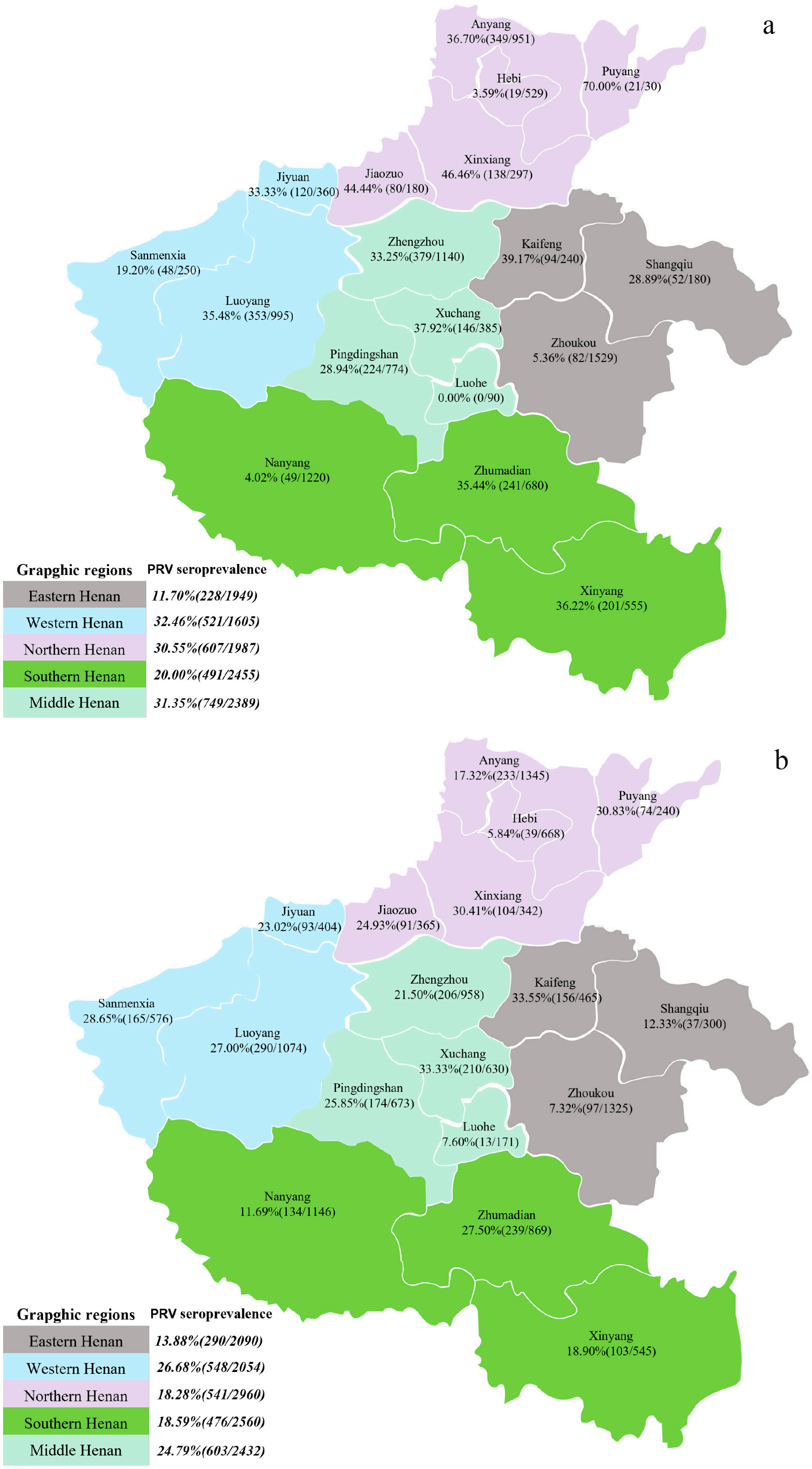
a
Anyang
36.70%(349/951)
Hebi
3.59%(19/529)
Puyang
70.00% (21/30)
Xinxiang
46.46% (138/297)
Jiaozuo
44.44% (80/180)
Jiyuan
33.33% (120/360)
Zhengzhou
33.25%(379/1140)
Kaifeng
39.17%(94/240)
Shangqiu
28.89%(52/180)
Sanmenxia
19.20% (48/250)
Luoyang
35.48% (353/995)
Xuchang
37.92%(146/385)
Zhoukou
5.36% (82/1529)
Pingdingshan
28.94%(224/774)
Luohe
0.00% (0/90)
Nanyang
4.02% (49/1220)
Zhumadian
35.44% (241/680)
Xinyang
36.22% (201/555)
Grapghic regions
PRV seroprevalence
Eastern Henan
11.70%(228/1949)
Western Henan
32.46%(521/1605)
Northern Henan
30.55%(607/1987)
Southern Henan
20.00%(491/2455)
Middle Henan
31.35%(749/2389)
b
Anyang
17.32%(233/1345)
Puyang
30.83%(74/240)
Hebi
5.84%(39/668)
Xinxiang
30.41%(104/342)
Jiyuan
23.02%(93/404)
Jiaozuo
24.93%(91/365)
Zhengzhou
21.50%(206/958)
Kaifeng
33.55%(156/465)
Shangqiu
12.33%(37/300)
Sanmenxia
28.65%(165/576)
Luoyang
27.00%(290/1074)
Xuchang
33.33%(210/630)
Pingdingshan
25.85%(174/673)
Zhoukou
7.32%(97/1325)
Luohe
7.60%(13/171)
Nanyang
11.69%(134/1146)
Zhumadian
27.50%(239/869)
Xinyang
18.90%(103/545)
Grapghic regions
PRV seroprevalence
Eastern Henan
13.88%(290/2090)
Western Henan
26.68%(548/2054)
Northern Henan
18.28%(541/2960)
Southern Henan
18.59%(476/2560)
Middle Henan
24.79%(603/2432)

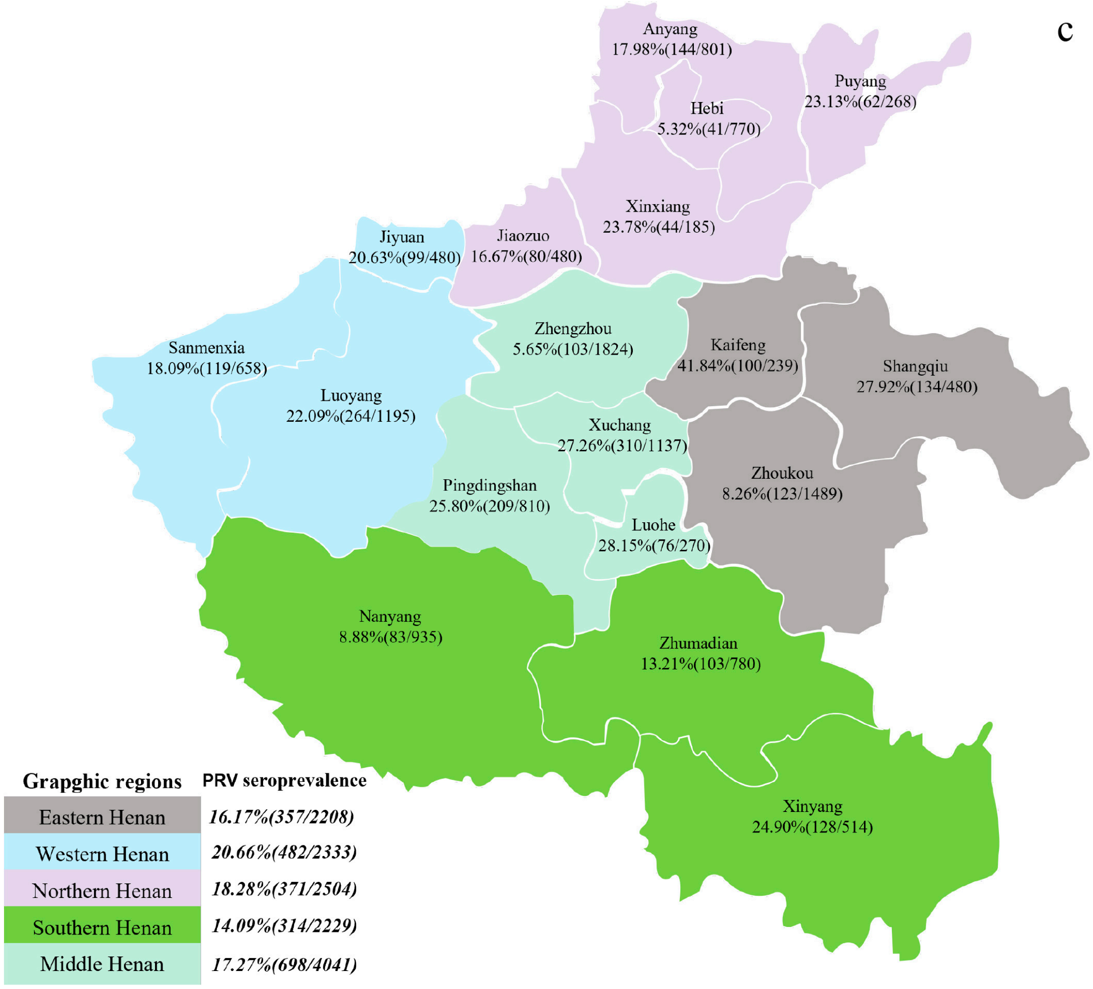

Figure S1. Seroprevalence of pseudorabies virus (PRV) in geographical distributions in Henan province of China from January to December. (a) Seroprevalence in 2019. (b) Seroprevalence in 2020. (c) Seroprevalence in 2021.
