# Supplementary material for: Serological Investigation and Genetic Characteristics of Pseudorabies Virus between 2019 and 2021 in Henan Province of China
Source: Viruses. 2022 Jul 30;14(8):1685. doi: 10.3390/v14081685 (PMC9412869; doi:10.3390/v14081685)
Supplement: Supplementary file 1 [file viruses-14-01685-s001.zip › Table S1.pdf]

Table S1. The information of PRV strains isolated in this study and reference strains for sequence alignment and phylogenetic analysis

Strain	Species	Geographical location	Collection time	gE length (bp)	GenBank No
**HD-1**	Pig	Handan, Hebei	2017	1740	MF409395
**HD-2**	Pig	Handan, Hebei	2017	1740	MF409396
**PY-1**	Pig	Puyang, Henan	2018	1740	MW073101
**SC1**	Pig	Wenshui, Shanxi	2018	1740	MH507059
**SC2**	Pig	Wenshui, Shanxi	2018	1740	ON602708
**HN-CM**	Pig	Kaifeng, Henan	2019	1737	ON602709
**HN-CY**	Pig	Jiaozuo, Henan	2019	1737	ON602710
**HN-GM**	Pig	Zhumadian, Henan	2019	1737	ON602711
**HN-HY**	Pig	Puyang, Henan	2019	1737	ON602713
**HN-LL**	Pig	Luoyang, Henan	2020	1737	ON602715
**HN-LH**	Pig	Luoyang, Henan	2020	1737	ON602714
**HN-LY**	Pig	Luoyang, Henan	2020	1737	ON602716
**HN-YH**	Pig	Xuchang, Henan	2021	1740	ON602720
**HN-HX**	Pig	Jiaozuo, Henan	2021	1737	ON602712
**HN-MY**	Pig	Sanmenxia, Henan	2021	1737	ON602717
**HN-WZ**	Pig	Puyang, Henan	2021	1737	ON602718
**HN-XT**	Pig	Jiaozuo, Henan	2021	1737	ON602719
**HN-YY**	Pig	Sanmenxia, Henan	2021	1737	ON602721
Fa	Cow	China	1962	1674	AF403049
SC	Pig	China	1986	1737	KT809429
Ea	Pig	China	1999	1737	AF171937
JS-2012	Pig	China	2012	1740	KP257591
HeN1	Pig	China	2012	1740	KP098534
HN1201	Pig	China	2012	1740	KP722022
NY-2012	Pig	China	2012	1740	KF130883
TJ	Pig	China	2012	1740	KJ789182
ZJ01	Pig	China	2012	1743	KM061380
HLJ8	Pig	China	2013	1740	KT824771
Anhui-CZ33	Pig	China	2017	1739	MK934522
HeNLH	Pig	China	2017	1740	MT775883
HuN-YY	Pig	China	2018	1737	MZ501781
hSD-1	Human	China	2019	1740	MT468550
HuB17	Pig	China	2020	1737	MT949537
Kaplan	Pig	Hungary	1959	1734	JF797218
NIA-1	Pig	Northern Ireland	1962	1734	FJ605136
Becker	Pig	USA	1967	1734	JF797219
NS374	Pig	Belgium	1971	1731	FJ605135
75V19	Pig	Belgium	1975	1734	FJ605133
89V87	Pig	Belgium	1989	1734	FJ605134
Yangsan	Pig	South Korea	1996	1734	AY249861
00V72	Pig	Belgium	2000	1734	FJ605132
P-PrV	Pig	Malaysia	2003	1737	FJ176390
Hercules	Pig	Greece	2010	1734	KT983810
Kolchis	Pig	Greece	2010	1734	KT983811
RC1	Procyon lotor	Japan	2016	1725	LC342744
NIA3	Pig	UK	2016	1734	KU900059

Note: Strains are represented at the front of the table
